# Unusual Length of Pedicle: Pedunculated Squamous Papilloma of Uvula Causing Unusual Dysphagia of Long Duration in a Child of 10 Years

**DOI:** 10.1155/2014/313506

**Published:** 2014-01-21

**Authors:** Ramisetty Sabitha Devi, B. Rajsekhar, G. Vijay Srinivas, Ninad J. Moon

**Affiliations:** ^1^Department of Oral Pathology, St. Joseph Dental College, Duggirala, Eluru, Andhra Pradesh, India; ^2^GEMS Medical College, Srikakulam, Andhra Prodesh, India; ^3^Oral Pathology, St. Joseph Dental College, Eluru, Andhra Prodesh, India; ^4^Department of Periodontics & Implantology, RKDF Dental College & Research Centre Bhopal, Madhya Pradesh, India

## Abstract

Benign oropharyngeal tumors are far less common compared to malignant tumors. Oropharyngeal papilloma is present in adults. Squamous papillomas are exophytic masses of oral cavity. When they occur on the palate they are most often asymptomatic and benign. Pedunculated squamous papillomas usually arise from the soft palate, tonsil, or the epiglottis. These lesions can sometimes prove to be fatal. A case of pedunculated squamous papilloma, arising from the laryngopharynx, the clinical presentation, the site of origin, and the size of the lesion were quite unusual. The narrow base of the pedicle made the intraoral excision possible. But here, we present a case of a 10-year-old boy who had history of dysphagia of 3-year duration for solid food and he was comfortable only in squatting position the reason being squamous papilloma of uvula (unusual site) atypically because of astonishing length of pedicle (2.3 inches).

## 1. Introduction

Benign oropharyngeal tumors are far less common compared to malignant tumors. Oropharyngeal papilloma is present in adults [1]. Squamous papillomas are exophytic masses of oral cavity. When they occur on the palate they are most often asymptomatic and benign [2]. Pedunculated squamous papillomas usually arise from the soft palate, tonsil, or the epiglottis. These lesions can sometimes prove to be fatal. A case of pedunculated squamous papilloma, arising from the laryngopharynx, the clinical presentation, the site of origin, and the size of the lesion were quite unusual. The narrow base of the pedicle made the intraoral excision possible.

## 2. Case Report

**Figure 1:**
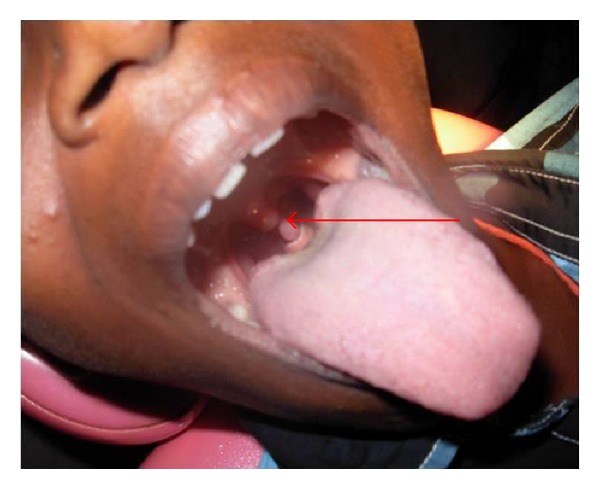
Clinical photo: arrow showing pedunculated squamous papilloma of uvula.

A 10-year old boy presented with 3 years of unusual dysphagia. He also reported choking sensation while eating solid food and was comfortable only in squatting position. He had a history of elongated pedunculated lesion. The patient had difficulty in swallowing food for 7 years because of local irritation in throat and dry cough. There was no relevant history of throat pain, fever, and any other infection. On throat examination extending from uvular tip a fine strand of tissue over 2.3 inches in length ending in a small disc-like mass-pedunculated (3 × 2 cms) basal part of uvula appeared to be normal. This caused difficulty in swallowing (in the laryngeal region). Excision was simple (see Figure 1).

**Figure 2:**
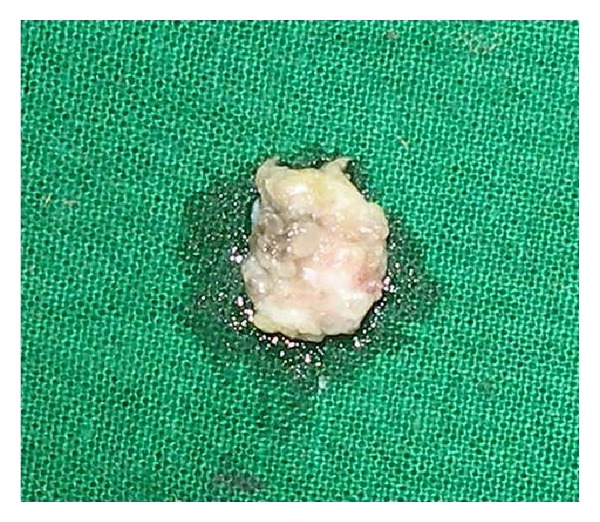
Macroscopic view of pedunculated squamous papilloma uvula.

*Grossly*. On examination the disc-like mass revealed rough and papillary projections giving a provisional diagnosis of papilloma (see Figure 2).

**Figure 3:**
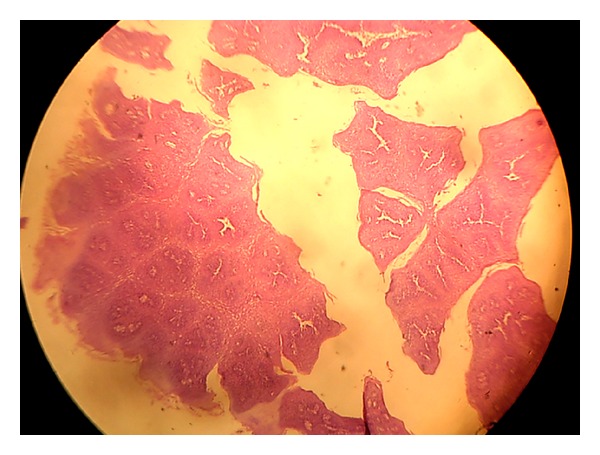
Microscopic view of pedunculated squamous papilloma uvula.

*Histopathological Examination*. Squamous papilloma is classically an exophytic lesion showing a complex pattern of finger-like projections with a central vascular zone surrounded by stratified squamous epithelium. Multiple papillary folds are hyperparakeratotic and epithelium also revealed plenty of koilocytes (see Figure 3).

## 3. Discussion

Pedunculated squamous papillomas usually arise from the soft palate, tonsil, or the epiglottis. These lesions can sometimes prove to be fatal. A case of pedunculated squamous papilloma, arising from the laryngopharynx, the clinical presentation, the site of origin, and the size of the lesion were quite unusual. The narrow base of the pedicle made the intraoral excision possible [3].

Oral squamous papilloma (OSP) is a benign proliferation of the stratified squamous epithelium, which results in a papillary or verrucous exophytic mass. The lesions were softened/flaccid in 66.7% of cases and a pedunculated attachment was seen in 75% of the lesions [4].

Male to female ratio is 1 : 1.5. The mean age was 33 years, with the majority in the 2nd, 3rd, and 4th decades. The sites commonly affected by benign neoplasms were the palate, tongue, upper lip, and buccal mucosa, in a descending order. 4% of squamous papilloma was common benign oral soft tissue masses. The most common location for squamous papilloma was the palate and tongue [5].

Malignant oropharyngeal tumors are far more common compared to benign tumors. Oropharyngeal papilloma is typically present in adults. Benign oropharyngeal tumors are far less common compared to malignant tumors. Oropharyngeal papilloma is typically diagnosed most often in adults. The papilloma is usually solitary and enlarges to a maximum size of about 0.5 cm [5]. But in our case papilloma is in rare site-uvula, big in size (3 × 2 cms), long pedunculated strand measured 2.3 inches in length and unusual cause of dysphagia makes it sound rare.

In 464 oral squamous cell papillomas, 34.3% of cases were located on the palatal complex (hard, soft, and uvula), but only 4.2% of cases were located on the uvula. Papilloma was greater than one cm in length, which occurs in less than 25% of cases of oral papillomas. Therefore, it is probable that the greater the length of the uvular lesion, the rarely [6].

They are most often benign and asymptomatic. But in our case the patient presented with dysphagia-irritation of throat-atypical [2]. Squamous cell papilloma arose from the uvula, which presented as unusual. But in our case it arose from uvula causing unusual dysphagia. Both support rarity [7].

Pathogenesis is related to human papilloma virus, but there is controversy regarding its viral origin. In our case section shows koilocytes [8]. Average size is less than 1 cms and only 8% were 2 cms and many are 3 to 4 mms [9]. There is a fine strand of tissue more than 2 inches terminating disc-like papilloma. In our case also it matches parallel.

## 4. Conclusion

Squamous cell papilloma is a benign tumor with a rare entity arising on uvula which caused unusual dysphagia (symptomatic) of long duration, and patient was comfortable only in squatting position; it revealed fine strand of tissue measuring 2.3 inches terminating with a mass measuring 3 × 2 cms, papilloma, and on section it revealed presence of koilocytes. Hence with the above features it is a rare entity.

## References

[1] Wadhera R, Kalra V, Gulati SP, Ghai A (2012). A big solitary oropharyngeal papilloma in a child. *Egyptian Journal of Ear, Nose, Throat and Allied Sciences*.

[2] Lindsay Goodstein A, Khan A, Pinczewski J, Young VN (2012). Symptomatic squamous papilloma of the uvula: report of a case and review of the literature. *Case Report in Otolaryngology*.

[3] Desai S, Rajaratnam K (1989). Pedunculated squamous papilloma of the laryngopharynx. *Journal of Laryngology and Otology*.

[4] Carneiro TE, Marinho SA, Verli FD, Mesquita ATM, Lima NL, Miranda JL (2009). Oral squamous papilloma: clinical, histologic and immunohistochemical analyses. *Journal of Oral Science*.

[5] Al-Khateeb TH (2009). Benign oral masses in a northern jordanian population-a retrospective study. *The Open Dentistry Journal*.

[6] Abbey LM, Page DG, Sawyer DR (1980). The clinical and histopathologic features of a series of 464 oral squamous cell papillomas. *Oral Surgery Oral Medicine and Oral Pathology*.

[7] MacDonald-Jankowski DS (1990). A squamous cell papilloma as a cause of dysphagia and vomiting. *British Dental Journal*.

[8] Jaju PP, Suvarna PV, Desai RS (2010). Squamous papilloma: case report and review of literature. *International Journal of Oral Science*.

[9] Abou-Elhamd KA, Yaquoby M (2010). Soft palate papilloma: a report of 4 cases with review of literature. *The Saudi Journal of Oto-Rhino Laryngology Head and Neck Surgery*.

